# Titanium Dioxide Photocatalytic Polymerization of Acrylamide for Gel Electrophoresis (_TIP_PAGE) of Proteins and Structural Identification by Mass Spectrometry

**DOI:** 10.1038/srep20981

**Published:** 2016-02-11

**Authors:** Wenyang Zhang, Zhiwei Yuan, Lulu Huang, Jie Kang, Ruowei Jiang, Hongying Zhong

**Affiliations:** 1 Mass Spectrometry Center for Structural Identification of Biological Molecules and Precision Medicine, Key Laboratory of Pesticides and Chemical Biology, Ministry of Education, College of Chemistry, Central China Normal University, Wuhan, Hubei 430079, P. R. China

## Abstract

Polyacrylamide gel electrophoresis (PAGE) coupled with mass spectrometry has been well established for separating, identifying and quantifying protein mixtures from cell lines, tissues or other biological samples. The copolymerization process of acrylamide and bis-acrylamide is the key to mastering this powerful technique. In general, this is a vinyl addition reaction initiated by free radical-generating reagents such as ammonium persulfate (APS) and tetramethylethylenediamine (TEMED) under basic pH and degassing experimental condition. We report herein a photocatalytic polymerization approach that is based on photo-generated hydroxyl radicals with nanoparticles of titanium dioxide. It was shown that the polymerization process is greatly accelerated in acidic condition when ultraviolet light shots on the gel solution containing TiO_2_ nanoparticles without degassing. This feature makes it very useful in preparing Triton X-100 acid urea (TAU) gel that has been developed for separating basic proteins such as histones and variants in acidic experimental condition. Additionally, the presence of titanium dioxide in the gel not only improves mechanistic property of gels but also changes the migration pattern of different proteins that have different affinities to titanium dioxide.

Since the invention in last century, polyacrylamide gel electrophoresis remains the most powerful separation technique for proteomics^1^,^2^,^3^ so far. By using this technique, thousands of cellulous proteins can be simultaneously fractionated and displayed with unparalleled high resolution in a gel. Coupled to mass spectrometry, it becomes an indispensible tool for protein structural identification^4^,^5^,^6^. Polyacrylamide gels are usually formed by copolymerization of acrylamide and bis-acrylamide with ammonium persulfate (APS) and tetramethylethylenediamine (TEMED) as the initiator and catalyst. There are mainly three steps for such gel preparation. (A) Initiation of free radicals. Free radicals are generated from ammonium persulfate and TEMED accelerates the rate of formation of free radicals from ammonium persulfate. (B) Elongation of polymer chains. Resultant free radicals further convert acrylamide monomers to free radicals that can react with unactivated acrylamide monomers to start elongation of polymer chains. In the meanwhile, the elongating polymer chains are crosslinked with bis-acrylamide, resulting in the formation of characteristic pores. (C) Termination of polymerization cascade. As the polymerization reaction proceeds, acrylamide monomers are gradually consumed and polymer chain elongation stops. For all these reactions, the pH value of the gel solution should be kept between 8 and 9 in order for ammonium persulfate and TEMED to act efficiently. Additionally, because TEMED is prone to oxidative reaction which causes the loss of catalytic activity, the gel solution should also be degassed under a vacuum of 125 torr or better for at least 15 minutes at room temperature. The major challenge of this protocol is the analysis of basic proteins such as histones and variants in acidic condition under which it takes very long time to complete polymerization process.

Histones and variants represent a very important class of proteins that contribute to the encoding of epigenetic information and regulation of gene expression^7^,^8^,^9^. Because of their similar molecular masses and extensive post-translational modifications, the resolution of current sodium dodecyl sulfate polyacrylamide gel electrophoresis (SDS PAGE) is not enough for fine separation of these proteins. Considering on their insolubility in neutral and basic solution, acidic polyacrylamide gels with urea and Triton X-100 as additives have been developed to separate acid extracted histones and histone variants on the basis of differences in charges^10^,^11^,^12^. In this technique, proteins are denatured by Triton X-100 or urea without affecting the charge of proteins. Under acidic running condition, the charge of proteins is solely determined by the number of protonated groups. Different modification levels of acetylation and phosphorylation of histone isoforms are mostly favored by this approach because they greatly affect the migration of histones through reduction of the positive charge of a Lys or introduction of an additional negative charge respectively. Despite the great success, preparation of acidic polyacrylamide gels is still very difficult by using ammonium persulfate and TEMED as the initiator and catalyst for polymerization reaction in acidic condition. It usually takes more than 1 hour to complete gelation process. In particular, the mechanistic property of resultant gels is not strong due to incomplete chain elongation and crosslinking.

Photocatalytic production of electron-hole pairs in the aqueous suspension of titanium dioxide under ultraviolet irradiation has been reported^13^,^14^,^15^ for many years. It is well-established that superoxide (O_2_^−^) and hydroxyl radical (·OH) can be generated by O_2_ capture of photoexcited electrons in conduction band and hole oxidization of H_2_O respectively. More than that, semiconductor nanoparticles have been increasingly recognized to initiate polymerization process with light excitation^16^,^17^,^18^,^19^,^20^,^21^,^22^. The photopolymerization technique provides a new way to prepare polymer/inorganic nanocomposites. This work is aimed to investigate the polymerization reaction of acrylamide and bis-acrylamide initiated by hydroxyl radicals in acidic condition. Compared with Azo-based initiator such as VA-086 which is also used for introducing hydroxyl group into terminal of polymer^23^,^24^,^25^, semiconductor nanoparticles not only speed up the polymerization process under light irradiation but also improve the mechanical properties because of the formation of cross-linked nanocomposites. The so called _TIP_PAGE approach (**ti**tanium dioxide **p**hotocatalytic **p**olymerization of **a**crylamide for **g**el **e**lectrophoresis) should be able to provide an alternative approach for proteomics.

## Experimental Section

### Preparation of Triton-Acid-Urea (TAU) Polyacrylamide Gel by Titanium Dioxide Photocatalytic Polymerization Reaction

All chemicals and apparatus are listed in Supplementary Notes 1. The components of separating gel and stacking gel were listed in Supplementary Table 1. The stacking gel was prepared by using ammonium persulfate and TEMED as the initiator and catalyst. As for the separating gel, nanoparticles of titanium dioxide were mixed with other chemicals after they were completely dissolved in water. The mixture was gently stirred until a uniform suspension of titanium dioxide was formed and polymerization process started. Air bubbles were carefully removed with a pipette. Then the mixture was gently poured to the gel casting model to 3 cm below top of plate and pure water was overlaid on the top. The separating gel was irradiated with ultraviolet light for 20 minutes to allow adequate polymerization. At room temperature (23~25 °C), gelation can complete in about 10 minutes. Additional 10 minutes is necessary to ensure full reaction. The stacking gel was casted on the top of the separating gel. After poured off water from the top of the polymerized separating gel, the stacking gel solution containing APS and TEMED was poured to 1.5 cm below the edge of the glass plate and a comb was inserted. Then pure water was overlaid on the top and it was allowed to polymerize for 1 hour. The gel should be made freshly right before downstream electrophoresis.

### Triton-Acid-Urea (TAU) Gel Electrophoresis with Titanium Dioxide Photocatalytically Polymerized Acrylamide Gel

In order to remove un-reacted residues, it is recommended to pre-run the freshly prepared gel at 100 volts for at least 1 hour. Histones were extracted with H_2_SO_4_ as previously reported^26^. Lyophilized histone powder (3 mg) or standard casein/de-phosphorylated casein was dissolved in 600 μl acidic sample buffer containing 0.36 g urea, 50 μl glacial acetic acid and 30 μL 40 mg/ml CuSO_4_ as well as 60 μl 0.2% Pyronin Y. CuSO_4_ was added to the sample buffer so that to chelate with histones and improve separation resolution^26^. Pyronin Y was used a marker to visualize running front line. Freshly prepared separating gel and stacking gel are installed in a mini gel system for electrophoresis experiments. About 50 μg histone proteins (10 μl sample buffer) or 10 μg standard casein/de-phosphorylated casein (2 μl sample buffer) were loaded into each lane. The running buffer contains 5% acetic acid solution. In order to concentrate the sample, first set the work voltage as 30 Volts and allow the gel to run for about 45 minutes. When the red dye pyronin Y was condensed into a straight line at the bottom of the stacking gel, increase the work voltage to 100 Volts and allow it to run for about 200 minutes. Finally, the TAU gel was stained as described in Supplementary Notes 2. Mass spectrometric identification of gel separated proteins was performed with a Waters MALDI Synapt G2 HDMS system (MA, USA) in positive ion and sensitivity mode as described in Supplementary Notes 3.

## Results and Discussion

### Chemical Polymerization Versus Photochemical Polymerization

**Figure 1:**
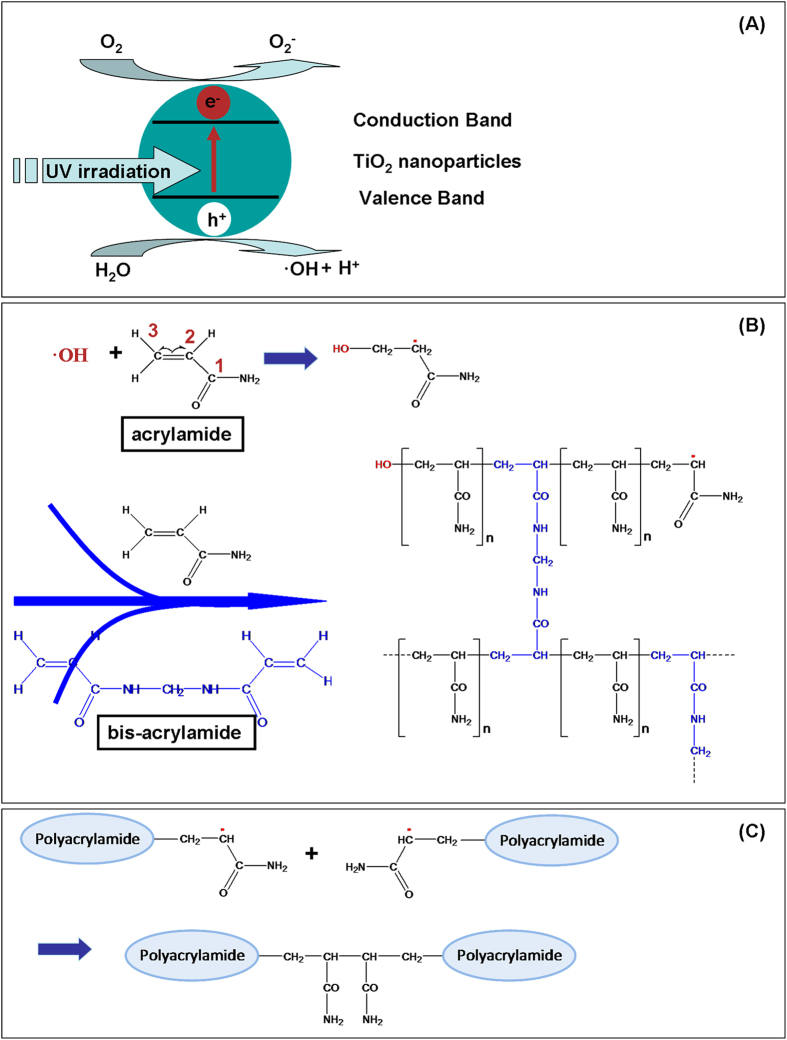
Mechanism of photochemical polymerization of acrylamide. (**A**) Photocatalytic generation of hydroxyl radicals on titanium dioxide nanoparticles. (**B**) Elongation of polymer chains and crosslinking with bis-acrylamide monomers. (**C**) Termination of the polymerization reaction.

As shown in Supplementary Fig. 1(A–C), ammonium persulfate and TEMED start the free radical reaction cascade. All three steps including initiation, elongation and termination are required to react in basic pH gel forming solution at which initiators are effective. The free base form of TEMED is essential for initiation reaction. At low pH, TEMED may become protonated, resulting in very slow polymerization. Careful examination of these reactions also reveals that the presence of oxygen may cause oxidization of TEMED and inhibition of polymerization reaction. Instead, the mechanism of photochemical polymerization was shown in Fig. 1. With ultraviolet irradiation, hydroxyl radicals are readily generated as shown in Fig. 1(A). Hydroxyl radical is a highly chemical reactive species which forms an important part of radical chemistry. Its role in pollutant degradation has been extensively investigated^26^,^27^,^28^. This work demonstrates the polymerization reaction cascade initiated by hydroxyl radicals. As shown in Fig. 1(B), there are three carbon atoms present in acrylamide monomer molecules. Charge distribution in this molecule is calculated in Supplementary Notes 4 and shown in Supplementary Fig. 2. Clearly, the 3^rd^ carbon atom has relatively higher electron density and less steric effect than other atoms. It becomes the most favorable site for photo-generated hydroxyl radicals to attack. Similar as that described in chemical polymerization, acrylamide monomers are converted into free radicals that can proceed to react with unactivated acrylamide monomers and the photochemical polymerization reaction cascade is started. In the meanwhile, double bonds of bis-acrylamide can also react with free radicals. The competition between hydroxyl radicals and resultant acrylamide radicals depends on the concentration of acrylamide monomers and bis monomers. Because bis acrylamide is present in much smaller quantities than acrylamide monomer, hydroxyl radicals mainly react with acrylamide monomers. Characteristic pores are formed when the elongating polymer chains are crosslinked with bis-acrylamide. Termination of the polymerization cascade was shown in Fig. 1(C). This photochemical polymerization approach avoids the use of ammonium persulfate and TEMED. In addition, photo-generated electrons are captured by oxygen in air, which prevents oxygen to trap free radicals and thus prevent it to inhibit acrylamide polymerization. Therefore this titanium dioxide photocatalytic polymerization of acrylamide for gel electrophoresis (_TIP_PAGE) approach can be used in acidic condition without degassing. It should be mentioned that acidic condition herein favors the photopolymerization reaction. In basic condition used for SDS PAGE experiments, titanium dioxide photocatalytic polymerization of acrylamide can not be observed. Additionally, although ribofalvin (riboflavin-5′-phosphate) has ever been developed as an initiator for photochemical polymerization reaction, it still needs to work with ammonium persulfate or TEMED^29^. The _TIP_PAGE approach makes use of the photocatalytic properties of semiconductors that are highly active in acidic condition.

### Electrophoretic Migration of Different Proteins on Titanium Dioxide Containing TAU Gel

**Figure 2:**
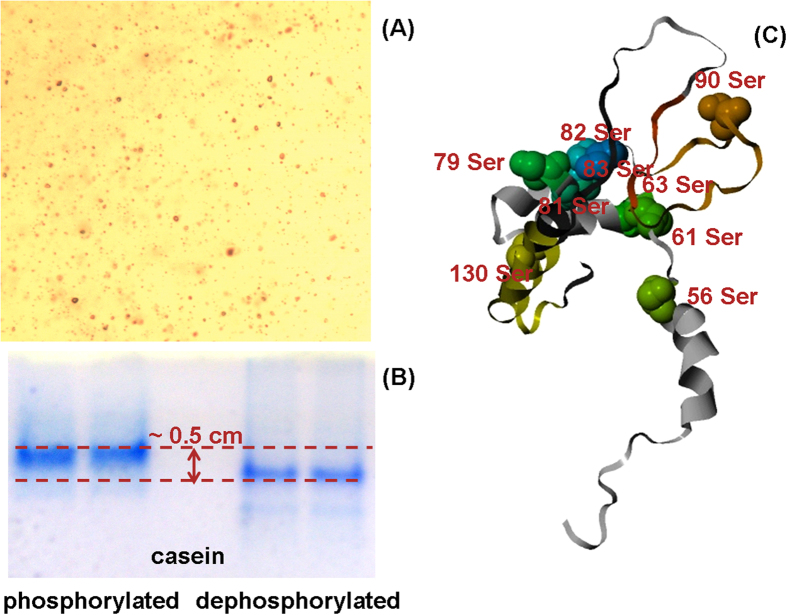
Electrophoretic migration of phosphorylated casein S1 and de-phosphorylated casein S1 proteins on titanium dioxide contained TAU gel. (**A**) Optical image of titanium dioxide contained TAU gel with 100 X magnification. (**B**) Gel image of phosphorylated casein and de-phosphorylated casein proteins on titanium dioxide contained TAU gel. (**C**) 3D structural modeling of phosphorylated casein S1 protein.

Due to protonation and oxidization of TEMED, the polymerization process of acrylamide in acidic condition is not only very slow but also generates mechanistically weak gels. The presence of titanium dioxide nanoparticles provides a substrate to differentially adsorb proteins with different affinities. In addition to photocatalytic activities, it has been found that TiO_2_ can specifically bind with phosphate groups^30^,^31^,^32^ and glycosylation groups^33^,^34^,^35^ of proteins. It thus should strongly affect electrophoretic migration of phosphorylated and non-phosphorylated proteins. Figure 2(A,B) are optical images of TAU gel with TiO_2_ nanoparticles under 100 X magnification and electrophoresis of phosphorylated and dephosphorylated casein proteins respectively. If the effects of TiO_2_, viscosity of the medium, differences in protein conformation and other experimental conditions are ignored, the migration rate differences between these two proteins can be simplified as the equation (1). Equation development was shown in Supplementary Notes 5.


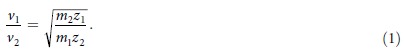


In the case of casein proteins whose molecular weights are more than 20 KDa, mass differences of phosphorylated or non-phosphorylated proteins do not have too much impact on their migration velocities. Essentially, charge Z determines the migration velocity 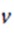. For α-S1 casein, there are 21 lysine or arginine basic residues in total, 9 phosphorylation sites and 720 Da mass differences. The mass ratio of phosphorylated over dephosphorylated casein is about 1.03. But the charge ration of these two proteins is about 0.57 because the presence of negatively charged phosphate groups neutralizes some of the positive charges carrying on protonated lysine and arginine residues. The expected velocity ratio of phosphorylated casein over dephosphorylated casein is about 0.75. Increased electrophoretic time should result in increased differences in migration distances in the gel. When TiO_2_ nanoparticles are present in the gel, electrophoretic migration velocity of phosphorylated proteins should be even slower than that of dephosphorylated proteins because of the binding of TiO_2_ with exposed negatively charged phosphate groups. As show in Fig. 2, after 200 minutes of electrophoresis with 100 volts of electrophoretic voltage, there are only about 0.5 cm differences (about 25% of the total, similar to that expected by eq. 1) in the migration distances of phosphorylated and dephosphorylated casein proteins. It seems that the effect of TiO_2_ binding contributes little to the migration in this case. Looking at the 3D structural model as shown in Fig. 2(C), because no detergents have been used to denature protein structures, some of the phosphate groups are hided in the folded protein structures, which prevent the interaction of TiO_2_ with phosphate groups. It was demonstrated that protein conformation actually plays important roles in acidic TAU gel electrophoresis.

The presence of titanium dioxide nanoparticles also strengthens the gels. As described in Supplementary Notes 6, formation of crosslinked organic/inorganic polymer nanocomposites greatly improves the elastic properties of gels. In the case of APS/TEMED catalyzed polymerization reaction, gels are always very soft and weak due to incomplete polymerization and cross-linking. It is difficult to transfer those gels further for the second dimensional separation. In contrast, TiO_2_ catalyzed polymerization reaction produces gels that can not only be easily transferred but also can be stretched almost twice of the original length. Additionally, the amount of titanium dioxide used for the gel does not show detectable effect on the sieving ability of the gel as shown in Supplementary Notes 7. The pore sizes of polyacrylamide gels usually range from several hundreds of nanometers (nm) to 70 nm. Sizes of nanoparticles of titanium dioxide used in this work are less than 25 nm as indicated by the manufacturers. As for nanoparticles which are much bigger or smaller than gel pores, they are washed away and do not affect the sieving ability of gels; As for nanoparticles whose sizes are equivalent to that of gel pores, they are trapped in the pores. So long as the amount of nanoparticles is in excess, it should be able to maintain the separation pattern of proteins when different amount of nanoparticles are used.

### Titanium Dioxide Photocatalytic Polymerization of Acrylamide for Gel Electrophoresis (_TIP_PAGE) of Acid Extracted Rat Liver Histone Proteins

**Figure 3:**
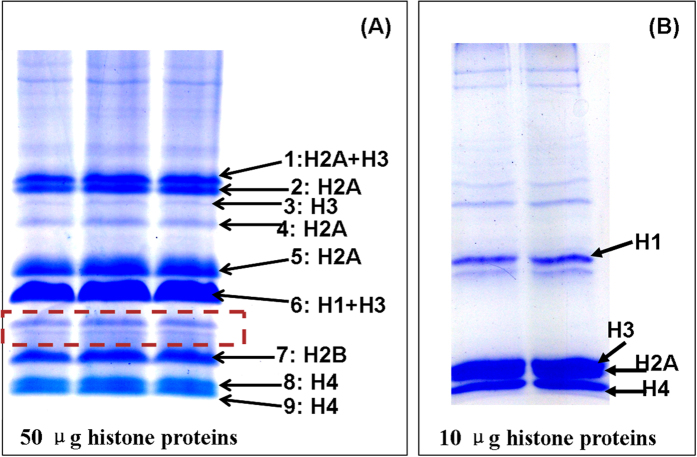
Analysis of acid extracted histones of male rat liver. (**A**) Gel image of histones with _TIP_PAGE approach. (**B**) Gel image of the same histone proteins with regular SDS PAGE approach.

Histone proteins from rat livers have been separated by using _TIP_PAGE approach. Fig. 3(A) is the gel image of these proteins. Compared with that of routine SDS PAGE shown in Fig. 3(B), it is not surprising that much more bands have been observed and several isoforms of histone proteins have been identified. All MS spectra of band 1–9 were shown in Supplementary 3–11 respectively. Compared with the same TAU gel electrophoresis without TiO_2_ that we reported previously^26^, the separation resolution of _TIP_PAGE has been greatly improved with finely dissected protein bands. For example, in TAU gel electrophoresis, H4 and H2B were co-eluted as one band. But with _TIP_PAGE approach, H2B and H4 have been significantly separated into at least 3 major bands (labeled as band 7, 8 and 9) with two H4 isoforms. The similar electrophoretic migration of H4 and H2B in TAU gel electrophoresis without TiO_2_ nanoparticles means these proteins have very similar mass-to-charge ratios. Changes in H2B mobility on TiO_2_ containing TAU gel may be ascribed to its posttranslational modifications. Examination of amino acid sequences of H2B and H4 shown in Supplementary Fig. 12 reveals that H2B have more than two times of possible phosphorylation sites than that of H4, which have strong affinities to TiO_2_ nanoparticles. Although only 3~4 phosphorylation sites among these have been reported so far, this work provides interesting experimental evidences for necessities of further investigation. Similar mass spectra of gel band 8 and 9 indicate that two isoforms of H4 have exactly the same tryptic peptides extracted from in-gel digestion. It means that different isoforms of H4 histone cannot be distinguished by this bottom-up proteomics approach. It can be proposed that the differential separation of H4 proteins by TiO_2_ TAU gel electrophoresis results from their differences in modifications. Attention should also be paid to the series bands just above the identified H2B band which was labeled with a dotted red square. These bands were not identified due to low abundance. But their absence in non-TiO_2_ TAU gel electrophoresis also indicates their possibilities for post-translational modifications. The separation of H2A and H3 isoforms has also been dramatically improved. Only one band for each H2A and H3 was observed in SDS PAGE. However, by using gels made with TiO_2_ photocatalytic polymerization reaction, H2A histones were detected in four different bands and H3 histones were detected in 3 different bands. In contrast, the separation of H1 was not significantly changed probably because of its less modified structures. All identified peptides were listed in Supplementary Table 2. MS/MS spectra as well as database searching results were shown in Supplementary Figs 13–22. Because of enhanced mechanical properties, _TIP_PAGE is highly reproducible for separation of histone proteins in acidic condition as shown in Supplementary Notes 8. Although the distribution of nanoparticles is not very uniform with 100 X magnification as shown in Fig. 2(A), it does not show obvious effects on protein separation.

## Conclusion

With UV irradiation, hydroxyl radicals generated on TiO_2_ nanoparticles can rapidly initiate polymerization reaction cascade of acrylamide without ammonium persulfate and TEMED. Hydroxyl radicals are highly reactive due to the presence of an unpaired electron. This photochemical polymerization starts as the attacking of hydroxyl radicals on the carbon atoms of acrylamide with high electron density but low steric effects. The polymer chain elongates through the reaction of resultant new free radicals with unactivated acrylamide monomers. Bis-acrylamide monomers are crosslinked with acrylamide polymer chains to form pores that can trap molecules with different sizes. TiO_2_ nanoparticles are not only involved in the photochemical reaction but also change the electrophoretic mobility of those proteins with phosphoryl modifications. The _TIP_PAGE approach is useful for separation of basic proteins such as histones in acidic condition.

## Additional Information

**How to cite this article**: Zhang, W. *et al*. Titanium Dioxide Photocatalytic Polymerization of Acrylamide for Gel Electrophoresis (_TIP_PAGE) of Proteins and Structural Identification by Mass Spectrometry. *Sci. Rep.* **6**, 20981; doi: 10.1038/srep20981 (2016).

## Supplementary Material

Supplementary Information
