# Supplementary material for: Titanium Dioxide Photocatalytic Polymerization of Acrylamide for Gel Electrophoresis (TIPPAGE) of Proteins and Structural Identification by Mass Spectrometry
Source: Sci Rep. 2016 Feb 11;6:20981. doi: 10.1038/srep20981 (PMC4750088; doi:10.1038/srep20981)
Supplement: Supplementary Information [file srep20981-s1.doc]

**Supporting Information:**

**Titanium Dioxide Photocatalytic Polymerization of Acrylamide for Gel Electrophoresis (_TIP_PAGE) of Proteins and Structural Identification by Mass Spectrometry**

**Wenyang Zhang, Zhiwei Yuan, Lulu Huang, Jie Kang, Ruowei Jiang, Hongying Zhong***

**Mass Spectrometry Center for Structural Identification of Biological Molecules and Precision Medicine**

**Key Laboratory of Pesticides and Chemical Biology, Ministry of Education, College of Chemistry, Central China Normal University, Wuhan, Hubei 430079, P. R. China**

*** To whom correspondence should be addressed. Email:** [**hyzhong@mail.ccnu.edu.cn**](mailto:hyzhong@mail.ccnu.edu.cn)

**1. Supplementary Figures**

**2. Supplementary Notes:**

**(1) Supplementary Notes 1**

**Chemicals and Apparatus.** LC-MS grade water, acetonitrile (ACN), methanol and Bradford protein assay reagent were purchased from Fisher Scientific (NJ, USA). Acrylamide, bis-acrylamide, ammonium persulfate (APS), N, N, N’, N’-tetramethylethylenediamine (TEMED), Coomassie Blue R-250, ammonium bicarbonate, H_2_SO_4_, CuSO_4_, sodium butyrate, NaN_3_, SDS (sodium dodecyl sulfate), Triton X-100, urea, ethanol, Tris-HCl and acetic acid of analytical reagent were purchased from Guoyao, China. Casein, dephosphorylated casein, trifluoroacetic acid (TFA), trichloroacetic acid (TCA), iodoacetamide, titanium dioxide nanoparticles (<25 nm), DTT (dithiothreitol), (Glu1)-Fibrinopeptide B and pyronin Y were purchased from Sigma-Aldrich (MO, USA). Sequence grade trypsin was purchased from Promega (WI, USA). C18 ZipTip was purchased from Millipore (MA, USA). PEG calibration standard was purchased from Waters (MA, USA). Cell lysis buffer was purchased from Shenergy Biocolor BioScience & Technology (Shanghai, China). Ultraviolet light (365 nm, 25 W, 200 x 50 mm) was purchased from Jiapeng Technology Co. Ltd. (Shanghai, China). MINI-gel EPS 300 system was purchase from Tanon (Shanghai, China). All gel images were taken with a HP scanner (HP, USA). The contrast of the images was set between 70 and 80 in order to clearly show the bands. Shaker TS-2 was purchased from Qilinbeier (Jiangsu, China). Male rats (Wistar) were purchased from Provincial Center for Experimental Animals of Hubei Province (Wuhan, China).

**(2) Supplementary Notes 2**

**Staining of Histone Proteins and Standard Casein/De-phosphorylated Casein.** The TAU gel was stained in a buffer containing methanol (50%) and Coomassie Blue R250 (0.1%) for about 1~2 hours with shaking. A destaining solution containing methanol (50%) and NH_4_HCO_3_ (5%) was used to remove back ground blue color of the gel. It takes about 1~2 hours to complete the destaining step with shaking. It should be indicated that the staining and destaining solutions should not contain acetic acid that is regularly used for SDS PAGE because basic histones maybe washed away. For comparison, the same batch of acid extracted histone proteins have been separated and stained by using routine SDS PAGE approach as previously reported^16^.

**(3) Supplementary Notes 3**

**Mass Spectrometric Identification of Gel Separated Proteins.** A Waters MALDI Synapt G2 HDMS system (MA, USA) in positive ion and sensitivity mode was used for all MS and MS/MS experiments. In addition to mass calibration with PEG standards, mass accuracy was ensured by using (Glu1)-Fibrinopeptide B as the lock-mass for real time instrumental calibration. The acquisition time for lock mass or each sample spot was 10 s and 60 s respectively. The laser energy was set as 230 units. Samples have been loaded into several lanes in parallel for repeated experiments in order to obtain as many as MS/MS spectra for improved identification of proteins. In-gel tryptic digestion was performed as that previously reported^16^. All spectra have been combined for MASCOT searching against NCBInr database and rattus taxonomy. Carbamidomethylation, oxidization (M) and phosphorylation (S, T and Y) were set as variable modifications. Error tolerances for peptides and MS/MS spectra were set as 30 ppm and 0.1 Da respectively. Additionally, assignment of different variants obtained from database searching has also been compared with that of literature reported in order to achieve unambiguous identification of highly similar histones.

**(4) Supplementary Notes 4**

**Density Functional Theory (DFT) Studies of Acrylamide Polymerization Initiated by Photo Generated Hydroxyl Radicals and Protein Structure Modeling.** Charge distribution of acrylamide was calculated by using Gauss View 5.0 software (Wallingford, CT, USA). Structure of the molecule was first optimized by using 3-21G basis set. Then the B3LYP functional was used to describe electron exchange and correlation, and the 6-31G^+^ (d) basis set was used to locate optimized ground-state and transition-state structures. Natural bond orbital (NBO) calculation was performed at the B3LYP/6-31G^+^(d) level in order to estimate the energy of molecules with the same geometry but in the absence of electronic delocalization.

In order to evaluate the binding of TiO_2_ with phosphate groups of casein proteins, 3D structural model of casein S1 (bovine) was obtained from Protein Model Portal (http://www.proteinmodelportal.org).

**(5) Supplementary Notes 5**

**Electrophoretic migration of two different proteins.** For two different proteins, equation 1 and 2 simplify the relationship between migration velocity v and mass-to-charge ratio when the magnitude of the electric field keeps the same E (V m^-1^), **if the effects of TiO_2_, viscosity of the medium, differences in protein conformation and other experimental conditions are ignored.**

$\frac{1}{2}m_{1}v_{1}^{2}=z_{1}E$ (1)

$\frac{1}{2}m_{2}v_{2}^{2}=z_{2}E$ (2)

Therefore the migration rate differences between these two proteins can be derived as the equation (3) shown as follow.

$\frac{v_{1}}{v_{2}}=\sqrt{\frac{m_{2}z_{1}}{m_{1}z_{2}}}$ (3)

For simplification, the effects of TiO_2_ particles, viscosity of the medium, differences in protein conformation and other experimental conditions are ignored. Under such situation, kinetic energy of a charged particle can be described as equation (1). The units for both sides are joules.

**(6) Supplementary Notes 6**

**Mechanical strength of TiO2 catalyzed polyacrylamide gels.**

It was found that gels prepared with titanium dioxide catalysis have very strong elastic properties. They can be stretched almost two times of the original length without breaking. As for the gels prepared with APS and TEMED catalysis in acidic condition, gels are very soft and easily broken due to incomplete gelation. They can only be stretched 1 cm longer than the original length. (In order to show clearly, gels were stained with Coomassie Blue R250).

**
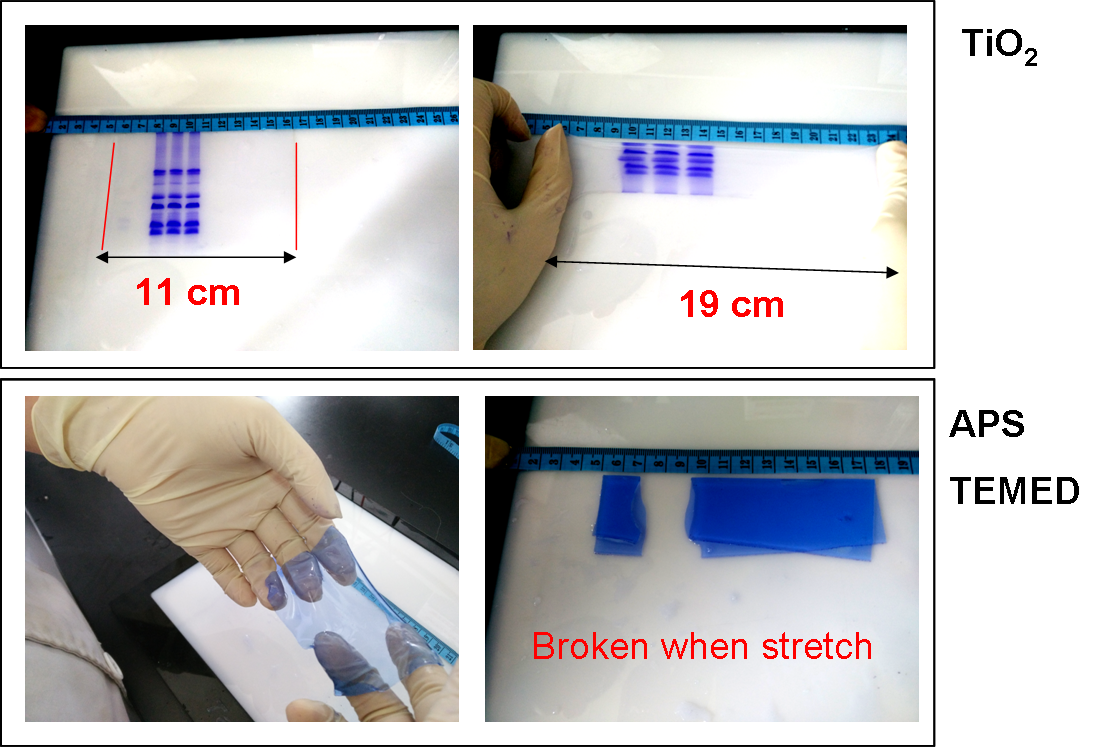
**

**Figure SN-1 Mechanical properties of gels made with different catalysts.**

Young’s module has been used to quantify the improvement of elasticity in TiO_2_ enhanced gel structure compared with non-TiO_2_ gel. The ratio of Young’s modulus of titanium dioxide catalyzed polyacrylamide gel over that of APS and TEMED catalyzed polyacrylamide gel can be calculated if the force applied to gels remains the same. According to Young’s modulus, the ratio of two different gels can be calculated as follows:

$$\frac{E_{TiO_{2}}}{E_{APS/TEMED}}=\frac{\Delta L_{APS/TEMED}}{\Delta L_{TiO_{2}}}=\frac{1}{8}$$

This result indicates that titanium dioxide photocatalytic polymerization of acrylamide produces very strong and elastic gels compared with non-titanium dioxide catalyzed polyacrylamide gels. The mechanical strength can be improved about 8 times by adding titanium dioxide nanoparticles into the gels.

**(7) Supplementary Notes 7**

**Effects of the amount of titanium dioxide nanoparticles used for the gel..**

**Figure SN-2. Separation of three different proteins with different molecular weights by using gels made with different amount of titanium dioxide nanoparticles.**

**(8) Supplementary Notes 8**

**Reproducibility demonstration of TiO_2_ catalyzed polyacrylamide gels.**

In order to demonstrate the reproducibility of proposed approach, rat liver histones have been repeatedly analyzed as shown in Figure SN-3.

**Figure SN-3. Reproducibility of TiO_2_ catalyzed polyacrylamide gels.**

| **Supplementary Table 1. Preparation of separating gel and stacking gel for mini-TAU gel electrophoresis (15% 60% : 0.4% acryl: bisacrylamide/6M urea/5% acetic acid) with _TOP_PAGE approach.** | | |
| --- | --- | --- |
| component | separating gel | stacking gel |
| Urea(g) | 3.6 | 1.8 |
| 60% : 0.4% acrylamide: bisacrylamide (ml) | 3.0 | 0.5 |
| Glacial acetic acid (µl) | 500 | 250 |
| dH_2_O (ml) | 3.73 | 2.8 |
| 10% Triton X-100 (µl) | 370 | ------ |
| N, N, N’, N’-tetramethylethylenediamine (µl) | ------ | 30 |
| 10% amonium persulfate (µl) | ------ | 140 |
| Nanoparticles of titanium dioxide (<25 nm) (mg) | 10 | ------ |

| **Supplementary Table 2. Identified tryptic peptides by using TiO_2_ photocatalytic polymerization of acrylamide for gel electrophoresis (_TIP_PAGE) of acid extracted histone proteins from rat livers.** | | | | | | | | |
| --- | --- | --- | --- | --- | --- | --- | --- | --- |
| No. | protein | gi | Mr (Expt.) | Mr (Cal.) | delta | score | E | sequences |
| 1 | histone H2A type 4 | 12025520 | 849.5237 | 849.5184 | 6.21 | 34 | 0.024 | HLQLAIR |
|  |  |  | 943.5307 | 943.5240 | 7.17 | 50 | 0.0022 | AGLQFPVGR |
|  |  |  | 1931.1797 | 1930.1615 | 528 | 46 | 0.00026 | VTIAQGGVLPNIQAVLLPK |
|  | histone H3.3B-like isoform 1 | 109463589 | 787.4763 | 787.4704 | 7.47 | 24 | 0.93 | KLPFQR |
|  |  |  | 1031.5941 | 1031.5876 | 6.32 | 29 | 0.23 | YRPGTVALR |
| 2 | Histone H2A | 66730355 | 849.5208 | 849.5184 | 2.80 | 37 | 0.012 | HLQLAIR |
|  |  |  | 943.5269 | 943.5240 | 3.15 | 50 | 0.0021 | AGLQFPVGR |
|  |  |  | 1931.1734 | 1930.1615 | 524 | 69 | 1.4e-006 | VTIAQGGVLPNIQAVLLPK |
| 3 | histone cluster 2, H3c2-like | 293342758 | 787.4723 | 787.4704 | 2.39 | 24 | 0.95 | KLPFQR |
| 4 | histone H2A.Z | 4504255 | 849.5256 | 849.5184 | 8.45 | 27 | 0.11 | HLQLAIR |
|  |  |  | 943.5309 | 943.5240 | 7.39 | 37 | 0.047 | AGLQFPVGR |
|  |  |  | 1950.0763 | 1949.0582 | 522 | 36 | 0.043 | HLQLAIRGDEELDSLIK |
| 5 | histone cluster 1, H2ak | 157821615 | 849.5186 | 849.5184 | 0.21 | 39 | 0.0056 | HLQLAIR |
|  |  |  | 943.5248 | 943.5240 | 0.92 | 45 | 0.0061 | AGLQFPVGR |
|  |  |  | 1691.8927 | 1691.8954 | -1.60 | 79 | 2.5e-006 | HLQLAIRNDEELNK |
|  |  |  | 1930.1615 | 1930.1615 | -0.00 | 52 | 5.1e-005 | VTIAQGGVLPNIQAVLLPK |
| 6 | histone (H1d) | 204595 | 1197.6649 | 1197.6605 | 3.70 | 50 | 0.0011 | ASGPPVSELITK |
|  |  |  | 1325.7602 | 1325.7554 | 3.60 | 35 | 0.027 | KASGPPVSELITK |
|  |  |  | 1578.7821 | 1577.7797 | 635 | 77 | 6.5e-006 | ALAAAGYDVEKNNSR |
|  | H3 histone, family 3B, isoform CRA_b | 149054821 | 787.4735 | 787.4704 | 3.91 | 18 | 2.5 | KLPFQR |
|  |  |  | 1031.5901 | 1031.5876 | 2.45 | 14 | 5.6 | YRPGTVALR |
| 7 | histone H2B type 1 | 12025526 | 1774.8057 | 1774.8018 | 2.19 | 100 | 1.1e-008 | AMGIMNSFVNDIFER.I + 2 Oxidation (M) |
| 8 and 9 | histone H4 replacement-like | 109504921 | 988.5707 | 988.5706 | 0.17 | 51 | 0.0014 | VFLENVIR |
|  |  |  | 1179.6128 | 1179.6135 | -0.59 | 65 | 5.2e-005 | ISGLIYEETR |
|  |  |  | 1324.7455 | 1324.7463 | -0.57 | 87 | 2.3e-007 | DNIQGITKPAIR |
